# An improved, scalable synthesis of Notum inhibitor LP-922056 using 1-chloro-1,2-benziodoxol-3-one as a superior electrophilic chlorinating agent

**DOI:** 10.3762/bjoc.15.271

**Published:** 2019-11-19

**Authors:** Nicky J Willis, Elliott D Bayle, George Papageorgiou, David Steadman, Benjamin N Atkinson, William Mahy, Paul V Fish

**Affiliations:** 1 Alzheimer’s Research UK UCL Drug Discovery Institute, The Cruciform Building, University College London, Gower Street, London WC1E 6BT, UK; 2 The Francis Crick Institute, 1 Midland Road, Kings Cross, London NW1 1AT, UK

**Keywords:** brain penetration, 1-chloro-1,2-benziodoxol-3-one, electrophilic chlorination, LP-922056, Notum inhibitor

## Abstract

**Background:** The carboxylesterase Notum has been shown to act as a key negative regulator of the Wnt signalling pathway by mediating the depalmitoleoylation of Wnt proteins. LP-922056 (**1**) is an orally active inhibitor of Notum. We are investigating the role of Notum in modulating Wnt signalling in the central nervous system and wished to establish if **1** would serve as a peripherally restricted control. An accessible and improved synthetic route would allow **1** to become more readily available as a chemical tool to explore the fundamental biology of Notum and build target validation to underpin new drug discovery programs.

**Results:** An improved, scalable synthesis of **1** is reported. Key modifications include: (1) the introduction of the C7-cyclopropyl group was most effectively achieved with a Suzuki–Miyaura cross-coupling reaction with MIDA-boronate **11** (**5** → **6**), and (2) C6 chlorination was performed with 1-chloro-1,2-benziodoxol-3-one (**12**) (**6** → **7**) as a mild and selective electrophilic chlorination agent. This 7-step route from **16** has been reliably performed on large scale to produce multigram quantities of **1** in good efficiency and high purity. Pharmacokinetic studies in mouse showed CNS penetration of **1** is very low with a brain/plasma concentration ratio of just 0.01. A small library of amides **17** were prepared from acid **1** to explore if **1** could be modified to deliver a CNS penetrant tool by capping off the acid as an amide. Although significant Notum inhibition activity could be achieved, none of these amides demonstrated the required combination of metabolic stability along with cell permeability without evidence of P-gp mediated efflux.

**Conclusion:** Mouse pharmacokinetic studies demonstrate that **1** is unsuitable for use in models of disease where brain penetration is an essential requirement of the compound but would be an ideal peripherally restricted control. These data will contribute to the understanding of drug levels of **1** to overlay with appropriate in vivo efficacy endpoints, i.e., the PK-PD relationship. The identification of a suitable analogue of **1** (or **17**) which combines Notum inhibition with CNS penetration would be a valuable chemical probe for investigating the role of Notum in disease models.

## Introduction

The Wnt signalling pathway has been shown to regulate crucial aspects of cell fate determination, organogenesis, cell migration and polarity [1]. Importantly, compromised Wnt signalling has been implicated in the perturbation of synaptic integrity and function in Alzheimer’s disease (AD) [2]. Palmitoleoylation of Wnt proteins is required for efficient binding to Frizzled receptors and the subsequent signal transduction. The carboxylesterase Notum has been shown to act as a key negative regulator of the Wnt signalling pathway by specifically mediating the depalmitoleoylation of Wnt proteins [3–4].

**Figure 1:**
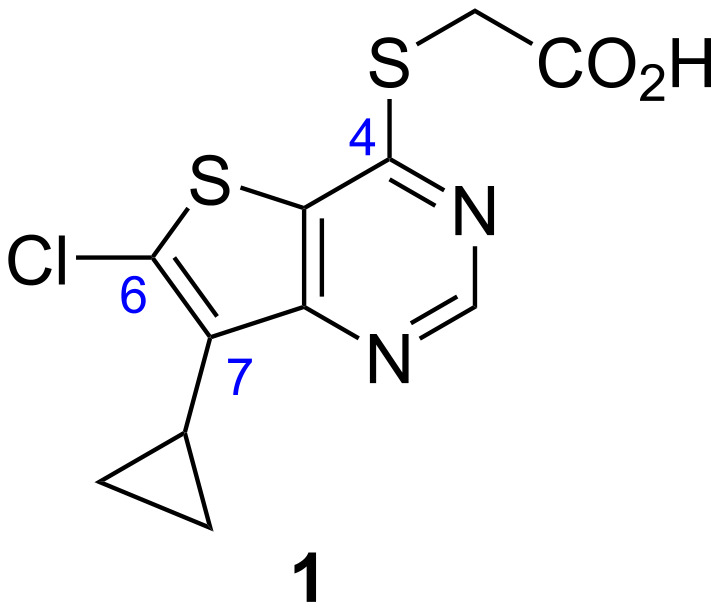
Chemical structure of Notum inhibitor LP-922056 (**1**).

LP-922056 (**1**, Figure 1) is an orally active inhibitor of Notum recently reported by Lexicon Pharmaceuticals [5–6]. Their research with **1** has shown that Notum is a potential drug target for stimulating bone formation and treating osteoporosis [7]. However, although **1** demonstrates low plasma clearance, the structure contains an essential carboxylic acid and acids tend to have low passive brain penetration [8–12]. We are investigating the role of Notum in modulating Wnt signalling in the central nervous system (CNS) [13] and wished to establish if **1** would serve as a peripherally restricted control compound. Hence, we required a synthetic route to **1** that could be reliably and safely performed on large scale.

A synthesis of **1** has been published in the patent literature [6], although many of the experimental procedures are described in terms of ‘general procedures’ which do not seem to work well when applied to **1** which contains functional groups sensitive to certain reagents employed (vide infra). An improved synthetic route should allow **1** to become more readily available as a chemical tool to explore the fundamental biology of Notum and build target validation to underpin new drug discovery programs for non-CNS disease.

## Results and Discussion

### Improved synthesis of **1**; first generation

**Scheme 1:**
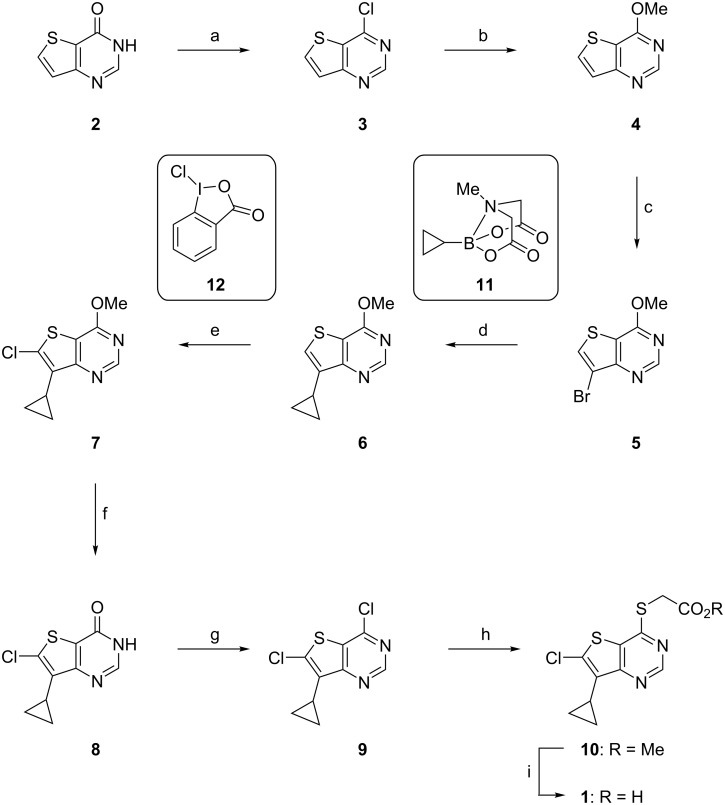
Synthesis of LP-922056 (**1**). Reagents and conditions^a^: (a) (COCl)_2_ (3.3 equiv), DMF, CH_2_Cl_2_, 55 °C , 16 h, 63–78%; (b) NaOMe (5 equiv), 1,4-dioxane, 0 °C then rt, 16 h, 92–93%; (c) NBS (1.1 equiv), AcOH/MeCN (1:100), 85 °C, 16 h, 41–48%; (d) **11** (1.5 equiv), Pd(PPh_3_)_2_Cl_2_ (5 mol %), K_3_PO_4_ (6 equiv), PhMe/H_2_O (3:1, 0.25 M), 100 °C, 16 h, 94–95%; (e) 1-chloro-1,2-benziodoxol-3-one (**12**, 1.5 equiv), DMF, 50 °C, 16 h, 77–94%; (f) HCl (12 M, 40 equiv), 70 °C, 16 h; (g) POCl_3_ (20 equiv), 90 °C, 16 h, 81% over 2 steps; (h) HSCH_2_CO_2_Me (**13**, 1.2 equiv), NEt_3_ (2.1 equiv), MeOH, 0 °C to rt, 16 h, 84%; (i) NaOH (1 M, 2 equiv), THF, 0 °C, 1 h, then HCl (1 M), 0 °C, 30 min, 98%. ^a^These reactions have been performed several times but have not been systematically optimised. Yields are the ranges obtained from repeated reactions. DMF, *N,N*-dimethylformamide; NBS, *N*-bromosuccinimide; THF, tetrahydrofuran.

Our first complete synthesis of **1** is presented in Scheme 1 (see Supporting Information File 1 for experimental procedures and characterisation data).

This short sequence starts with 4-chlorothieno[3,2-*d*]pyrimidine (**3**), which is readily available from commercial suppliers, and generally follows published procedures [5–6] but with key modifications to increase yields/selectivities and significantly improve ease of purification of key intermediates. Our modifications include: (1) the introduction of the C7-cyclopropyl group was most effectively achieved with a Suzuki–Miyaura cross-coupling reaction with MIDA-boronate **11** (**5** → **6**); and (2) C6 chlorination was performed with 1-chloro-1,2-benziodoxol-3-one (**12**) (**6** → **7**) as a mild selective electrophilic chlorination agent.

4-Chlorothieno[3,2-*d*]pyrimidine (**3**) was either purchased or prepared from thieno[3,2-*d*]pyrimidin-4(*3H*)-one (**2**) by C4 chlorination with oxalyl chloride/DMF following the method of Mitchell et al. [14]. Treatment of **3** with NaOMe displaced the C4–Cl to give **4** in a good yield as described by Atheral et al. [15]. Thieno[3,2-*d*]pyrimidine **4** is now suitably functionalised for the introduction of the C7-cyclopropyl group, the C6-chlorine atom and elaboration of the thioacetic acid moiety at C4.

Electrophilic bromination at C7 with *N*-bromosuccinimide gave **5** as the major regioisomer reproducibly on 100 mmol scale in modest yield (41–48%). This proved to be the least efficient step in our sequence and justified further optimisation (vide infra). Suzuki–Miyaura cross coupling of bromide **5** with cyclopropylboronic acid (2.5 equiv) produced **6** in good yield (62–89%) but the product required extensive chromatographic purification. We reasoned that switching from the boronic acid (*c*-PrB(OH)_2_) to the corresponding MIDA-boronate **11** would improve the quality of the reagent and slow release of the active boron species during the course of the reaction would allow us to reduce the number of molar equivalents required to improve conversion [16]. Palladium-mediated cross coupling of **5** with **11** (1.5 equiv) gave **6** in reproducibly high yield (ca. 95%) when performed on gram scale. However, when performed on larger scale (9.3 g), the reaction stalled after ca. 90% conversion and addition of extra catalyst Pd[(PPh_3_)_2_Cl_2_] and/or **11** failed to drive the reaction to completion. We found the most efficient way to complete the reaction conversion was to isolate the crude product (mostly **6**) and subject this material to a repeat reaction; this procedure gave **6** in a good yield 95% and simplified purification.

**Scheme 2:**
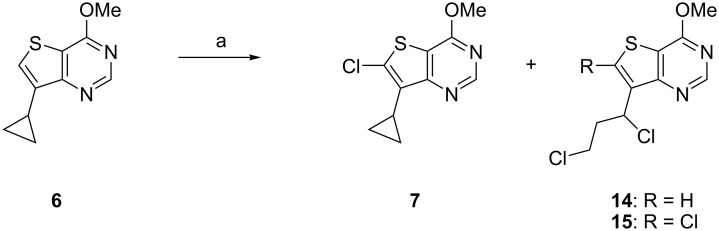
Chlorination of **6** with *N*-chlorosuccinimide (NCS). Reagents and conditions: (a) NCS (1.2 equiv), AcOH, 55 °C, 7 h, 15–32%.

With multigram quantities of **6** in hand, attention was turned to the C6 chlorination step. Unfortunately, despite this reaction being reported in the literature, there were no experimental details for this specific transformation as only a ‘general procedure’ was described [6]. Our attempts to use this procedure with *N*-chlorosuccinimide (NCS) as the chlorinating agent gave poor yields of the desired product **7** (ca. 15–32%) due to competing ring opening reactions of the 7-cyclopropyl group (Scheme 2). Clearly, a better procedure was required.

A large number of electrophilic chlorinating reagents for the direct chlorination of aromatic rings have been reported [17]. Recently, Xue et al. described the electrophilic chlorination of arenes and heterocycles by 1-chloro-1,2-benziodoxol-3-one (**12**) [18–19]. The hypervalent iodine(III) reagent **12** is reported to be a mild and effective reagent for the chlorination of nitrogen containing heterocycles which is easy to prepare and is air- and moisture-stable. The scope of published substrates includes chlorination of 7*H*-pyrrolo[2,3-*d*]pyrimidines and we wished to see if we could extend the scope to include sulphur containing heterocycles such as thieno[3,2-*d*]pyrimidines (e.g., **6**). It was also important to explore if **12** would efficiently chlorinate **6** at the less activated C6 position in the presence of the C7 cyclopropyl group.

Treatment of **6** with **12** (1.5 equiv) in DMF at 50 °C for 16–24 h gave the desired chloro product **7** in 77–94% isolated yield. Analysis of the crude reaction mixture showed only trace amounts of cyclopropyl ring opened products such as **14**/**15** as detectible by LC–MS. Hence, **12** proved to be a far superior reagent, when compared to NCS, for the C6 chlorination of thieno[3,2-*d*]pyrimidine **6** (i.e., **6** → **7**).

Completion of our synthesis of **1** followed established procedures although it proved expedient to carry material through several of these later steps without the need for extensive purification beyond a simple work-up procedure (**7** → **8** → **9**). Activation of C4 was accomplished by a two-step procedure of acid hydrolysis of the C4-OMe of **7** to give thieno[3,2-*d*]pyrimidin-4(3*H*)-one **8**, followed by chlorination with POCl_3_ to give **9**. Finally, nucleophilic displacement of the C4–Cl of **9** by methyl thioglycolate (**13**) gave ester **10** which was hydrolysed with NaOH to afford **1**. This route has been reliably performed on large scale to produce multigram quantities of **1** in good efficiency (total yield over 8-steps from **3**: 18–26%) and high purity (>99%).

### Improved synthesis of **1**; second generation

**Scheme 3:**
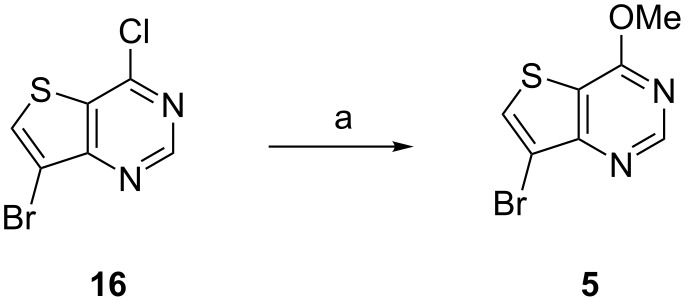
Improved synthesis of **5**. Reagents and conditions: (a) NaOMe (5 equiv), 1,4-dioxane, 0 °C then rt, 16 h, 84%.

A shorter synthesis was then developed by accessing bromide **5** by an alternative route. The low-yielding C7 bromination of **4** with NBS to give **5** as described above (Scheme 1, step c) was avoided by starting with 7-bromo-4-chlorothieno[3,2-*d*]pyrimidine (**16**) which is readily available from commercial suppliers. Treatment of **16** with NaOMe displaced the C4–Cl to give **5** in good yield on 10 g scale (Scheme 3). Even though **16** is somewhat more expensive than **2** or **3** per unit cost (by ca. 5-fold), this updated route shortens the sequence to just 7 steps from **16** and improves the overall yield to 40–50 %.

### Mouse pharmacokinetics for **1**

Assessment of **1** in mouse liver microsomes (MLM) showed excellent metabolic stability (Cl_i_ 1.0 μL/min/mg protein) which predicts for low clearance in vivo. Binding to mouse plasma proteins (mPPB) was very high with percent unbound drug (f_u_) of just 0.1%; this mPPB value can be used to calculate free drug concentrations from measured drug levels in plasma taken during in vivo experiments. The high mPPB is entirely consistent with the physicochemical properties of **1** as a lipophilic acid (mw 300; *c*Log*P* 3.1; *c*p*K*_a_ 3.1).

**Figure 2:**
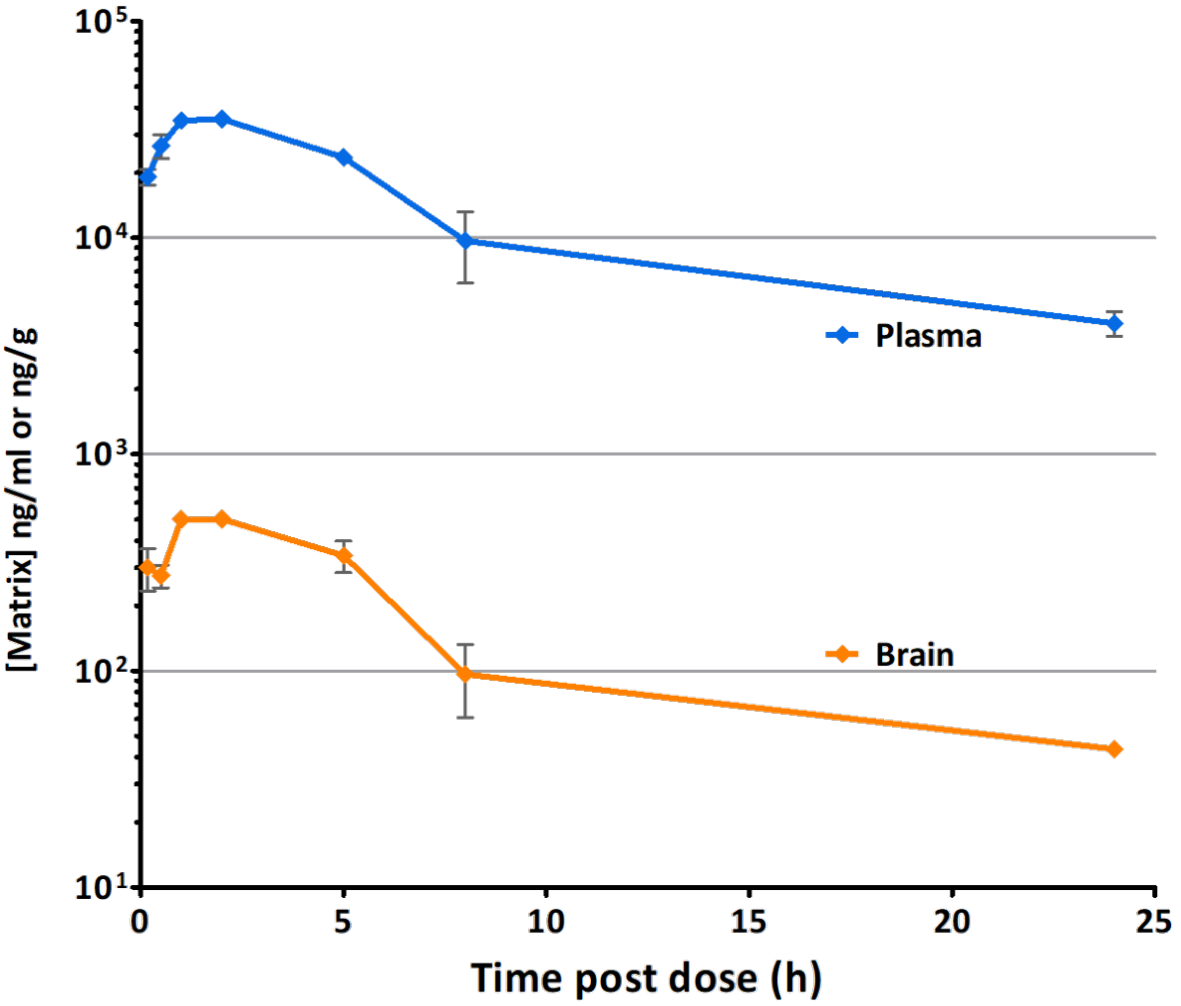
Concentrations of **1** in mouse following oral administration (p.o.) at 10 mg/kg.

Pharmacokinetic (PK) data for **1** was generated in vivo in mouse to evaluate brain penetration (Table 1; Figure 2) (see Supporting Information File 1, Tables S1–S3). The route of administration and dose were selected to most closely match relevant published mouse disease model studies [5,7]. Following single oral dose (p.o.) of 10 mg/kg, plasma exposure was high and plasma clearance was low relative to liver blood flow resulting in a plasma elimination half-life of 8.8 hours. The plasma parameters from these mouse PK experiments (*C*_max_ and AUC) are consistent with published preclinical PK data [5].

**Table 1:** Mouse pharmacokinetic data for **1**; oral (p.o.) dose at 10 mg/kg.^a^

PK Parameter	Plasma	Brain

*T*_1/2_	8.8 h	7.1 h
*T*_max_	2.0 h	2.0 h
*C*_max_	35,400 ng/mL	500 ng/g
AUC_0→t_	303,000 h·ng/mL	3,700 h·ng/g
AUC_0→∞_	354,000 h·ng/mL	4,080 h·ng/g

^a^Male fed CD1 mouse; suspension formulation in 0.1% Tween80 in water; *n* = 3 per time point; terminal blood and brain levels measured at seven time points: 0.17, 0.50, 1, 2, 5, 7.5 and 24 h.

CNS penetration of **1** was very low with a brain/plasma concentration ratio of ≈0.01 at all time points measured and was also 0.01 based on AUC_(0→∞)_. At this level of exposure, a significant proportion of the compound detected in brain samples is likely to have arisen from residual blood in the brain tissue.

### Amide analogues of **1** to explore CNS penetration and future opportunities

**Scheme 4:**
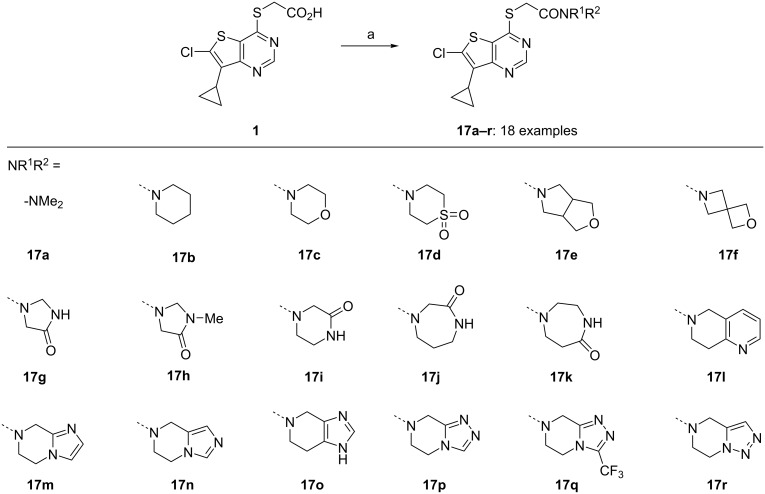
Preparation of amides **17**. Representative reagents and conditions^a^: (a) HBTU (1.1 equiv), iPr_2_NEt (2.5 equiv), DMF, rt, 15 min; then amine **18** (1.05 equiv). ^a^These reactions were performed once for each amide and have not been optimised. DMF, *N,N*-dimethylformamide; HBTU, *O*-(1*H*-benzotriazol-1-yl)-*N,N,N',N'*-tetramethyluronium hexafluorophosphate.

With a peripherally restricted control in hand, we elected to explore if **1** could be modified to deliver a CNS penetrant tool by capping off the acid as an amide. A small library of amides **17** were prepared from acid **1** by activation with HBTU and then subsequent reaction with the amine **18** (Scheme 4). These amides **17** were designed to have molecular properties (mw, *c*Log*P*, tPSA, HBD, p*K*_a_) consistent with CNS drug-like space [20].

Although significant Notum inhibition activity could be achieved (IC_50_ < 100 nM), none of these specific amides demonstrated the required combination of sufficient MLM stability along with cell permeability as measured by transit performance across a MDCK-MDR1 monolayer without evidence of P-gp mediated efflux. This collection of data for amides **17a**–**r** is shared as ‘open data’ to assist others in evaluating these results with the objective of solving this challenge (see Supporting Information File 1, Table S4 and Supporting Information File 2). Our own efforts took us to an alternative chemotype [21].

## Conclusion

An improved, scalable synthesis of Notum inhibitor **1** is reported. Key modifications include: (1) the introduction of the C7-cyclopropyl group was most effectively achieved with a Suzuki–Miyaura cross-coupling reaction with MIDA-boronate **11** (**5** → **6**); and (2) C6 chlorination was performed with 1-chloro-1,2-benziodoxol-3-one (**12**) (**6** → **7**) as a mild selective electrophilic chlorination agent. This 7-step route from **16** has been reliably performed on large scale to produce multigram quantities of **1** in good efficiency.

Pharmacokinetic studies in mouse showed CNS penetration of **1** is very low with brain/plasma concentration ratio of just 0.01 based on AUC_(0→∞)_. Hence, **1** is unsuitable for use in models of disease where brain penetration is an essential requirement of the compound but would be an ideal peripherally restricted control. These data will contribute to the understanding of drug levels of **1** to overlay with appropriate in vivo efficacy endpoints, i.e., the PK-PD relationship. The full PK data set is presented and shared as ‘open data’. This complete data set (along with others) will also assist with the creation of improved predictive pharmacokinetic models.

A small library of amides **17** were prepared from acid **1** to explore if **1** could be modified to deliver a CNS penetrant tool by capping off the acid as an amide. Although significant Notum inhibition activity could be achieved, none of these amides demonstrated the required combination of metabolic stability along with cell permeability without evidence of P-gp mediated efflux. The identification of a suitable analogue of **1** (or **17**) which combines Notum inhibition with CNS penetration would be a valuable chemical probe for investigating the role of Notum in disease models. This collection of data for amides **17a**–**r** is shared as ‘open data’ to assist others in evaluating these results with the objective of solving this challenge.

## Supporting Information

(1) experimental procedures and characterisation data for **3**–**10** and **1**; (2) mouse PK data which includes: study design summary; plasma concentrations; brain concentrations; (3) Notum IC_50_ (nM), MLM Cl_i_ (μL/min/mg protein) and MDCK-MDR1 AB/BA *P*_app_ (× 10^−6^ cm/s) data for **17a**–**r**.

To view the NMR spectra, use the file within the pdata folder of Supporting Information File 3.

File 1 Experimental section, mouse pharmacokinetics and profiles of amides.

File 2 Profiles of amides.

File 3 Raw NMR data files for compound LP-922056.
