# Supplementary material for: An improved, scalable synthesis of Notum inhibitor LP-922056 using 1-chloro-1,2-benziodoxol-3-one as a superior electrophilic chlorinating agent
Source: Beilstein J Org Chem. 2019 Nov 19;15:2790–7. doi: 10.3762/bjoc.15.271 (PMC6880826; doi:10.3762/bjoc.15.271)
Supplement: File 1 — Experimental section, mouse pharmacokinetics and profiles of amides. [file Beilstein_J_Org_Chem-15-2790-s001.pdf]

BEILSTEIN JOURNAL OF ORGANIC CHEMISTRY

Supporting Information

for

An improved, scalable synthesis of Notum inhibitor LP-922056 using 1-chloro-1,2-benziodoxol-3-one as a superior electrophilic chlorinating agent

Nicky J. Willis, Elliott D. Bayle, George Papageorgiou, David Steadman, Benjamin N. Atkinson, William Mahy and Paul V. Fish

Beilstein J. Org. Chem. **2019,** *15,* 2790–2797. doi:10.3762/bjoc.15.271

Experimental section, mouse pharmacokinetics and profiles of amides

License and Terms: This is a supporting information file under the terms of the Creative Commons Attribution License (http://creativecommons.org/licenses/by/4.0). Please note that the reuse, redistribution and reproduction in particular requires that the authors and source are credited. The license is subject to the *Beilstein Journal of Organic Chemistry* terms and conditions: (https://www.beilstein-journals.org/bjoc)

Table of contents:

Page S2 Experimental procedures and characterisation data for **3**-**10** and **1**.

Page S8 Animal testing declaration

Page S8 Table S1: Mouse pharmacokinetics - study design summary

Page S9 Table S2: Mouse pharmacokinetics - plasma concentrations

Page S10 Table S3: Mouse pharmacokinetics - brain concentrations

Page S11 Table S4: Profiles of amides **17a–r** - Notum IC_{50} (nM), MLM Cl_i (μL/min/mg protein) and MDCK-MDR1 AB/BA P_{app} ($\times 10^{-6}$ cm/s)

Page S13 SI References

Experimental section

General methods:

Unless preparative details are provided, all reagents were purchased from commercial suppliers and used without further purification. Anhydrous solvents were purchased as such from Acros Organics or Sigma Aldrich. Thin layer chromatography (TLC) was carried out on aluminium backed silica plates. The plates were visualized under UV (254 nm) light, followed by staining with phosphomolybdic acid dip or potassium permanganate and gentle heating. Compound purification by column chromatography was performed with a Biotage Isolera using prepacked Biotage SNAP KP-Sil silica cartridges or Biotage SNAP Ultra C18 reverse phase cartridges. Organic solvent layers were routinely dried with anhydrous $MgSO_4$ or Na_2SO_4 and concentrated using a Büchi rotary evaporator. Aqueous mixtures were dried on a VirTis BenchTop Pro freeze dryer.

Melting points were determined using Stuart SMP20 melting point equipment using closed end glass capillary tubes and are uncorrected. Infrared spectra were recorded on a Shimadzu IRTracer-100 FT-IR spectrometer, using a Universal ATR accessory for sampling, with absorbance (ν_{max}) reported in cm^{-1}. ^{1}H NMR / ^{13}C NMR spectra were performed in deuterated (≥ 99.5%) solvents on either a Bruker Avance 600 (600 MHz) or Bruker Avance 700 (700 MHz). Chemical shifts (δ) are reported as parts per million (ppm), coupling constants (*J*) are reported in Hz and signal multiplicities are reported as singlet (s), doublet (d), triplet (t), quartet (q), pentet (p), quintet (qu), sextet (sext), doublet of doublets (dd), doublet of triplets (dt), triplet of triplets (tt), multiplet (m), or broad singlet (br s). LCMS analysis was performed on a Waters Acquity H-Class UPLC system with either an acidic (HSS C18 Column, H_2O/acetonitrile, 0.1% formic acid) or basic (BEH C18 Column, H_2O/acetonitrile, 10 mM NH_4OH) mobile phase. HRMS data acquisition was achieved on a Waters Micromass LCT Premier electrospray time-of-flight (ESI-TOF) mass spectrometer. The observed mass and isotope patterns matched the corresponding theoretical values as calculated from the expected elemental formula.

The experimental procedures for the preparation of **3**, **4**, **8**, **9**, **10** and **1** generally followed published methods and characterisation data was consistent with that reported and the assigned structures.

*c*Log*P* and c*pK*$_a$ values were calculated with ChemDraw version 16.0.1.4 (77) software.

4-Chlorothieno[3,2-d]pyrimidine (**3**)

4-Chlorothieno[3,2-*d*]pyrimidine (**3**) was either purchased from Ark Pharm Inc. (AK-25196) or prepared from thieno[3,2-*d*]pyrimidin-4(*3H*)-one (**2**) by the method of Mitchell et al. [1].

4-Methoxythieno[3,2-d]pyrimidine (**4**)

Sodium methoxide (28.2 g, 521 mmol, 5.3 equiv) was added portionwise to a clear yellow solution of 4-chlorothieno[3,2-*d*]pyrimidine (**3**) (16.8 g, 98.5 mmol) in anhydrous 1,4-dioxane (300 mL) at 0 °C . The reaction mixture was stirred for 5 min, warmed to room temperature and then stirred for a further 16 h. The reaction mixture was concentrated under reduced pressure and the residue partitioned between ethyl acetate (250 mL) and water (50 mL). The organic phase was separated and the aqueous phase extracted with ethyl acetate (2 × 100 mL). The combined organic phase were dried (Na_2SO_4) and concentrated under reduced pressure to yield 4-methoxythieno[3,2-*d*]pyrimidine (**4**) (15.2 g, 91.5 mmol, 93%) as a light yellow solid that was used without further purification.

Mp 104-105 °C (lit 92-94 °C [2]); ^{1}H NMR (700 MHz, DMSO) δ 8.77 (s, 1H, C(2)H), 8.35 (d, $^3J_{HH}$ = 5.3 Hz, 1H, C(6)H), 7.59 (d, $^3J_{HH}$ = 5.3 Hz, 1H, C(7)H), 4.12 (s, 3H, OMe); ^{13}C NMR (176 MHz, DMSO) δ 163.91 (C(4)), 161.87 (C(7a)), 154.29 (C(2)), 136.09 (C(6)), 124.25 (C(7)), 116.74 (C(4a)), 54.17 (OMe); LCMS *m/z* (ESI$^+$) 167.1 ([M+H]$^+$, 100%); HRMS (ESI$^+$) $C_7H_6N_2OS^+$ ([M+H]$^+$) requires 167.0274; found 167.0271.

7-Bromo-4-methoxythieno[3,2-d]pyrimidine (**5**).

N-Bromosuccinimide (28.3 g, 158 mmol, 1.1 equiv) was added portionwise to a stirred solution of 4-methoxythieno[3,2-*d*]pyrimidine (**4**) (24.0 g, 144 mmol) in anhydrous acetonitrile (600 mL) and acetic acid (6 mL) and the reaction mixture was then heated at 85 °C for 16 h. The reaction mixture was cooled to room temperature, concentrated under reduced pressure, and partitioned between dichloromethane (250 mL) and brine (75 mL). The aqueous phase was further extracted with dichloromethane (150 mL), and the combined organics were dried (Na_2SO_4) and evaporated under reduced pressure. The crude material was purified by column chromatography on silica gel (5% ethyl acetate in cyclohexane) to give 7-bromo-4-methoxythieno[3,2-*d*]pyrimidine (**5**) (17.1 g, 69.8 mmol, 48%) as an off-white solid.

Mp 162-163 °C; ν_{max} (neat) 3053 w, 1566 m, 1518, 1458, 1417; ^{1}H NMR (700 MHz, DMSO-d_6) δ 8.86 (s, 1H, C(2)H), 8.54 (s, 1H, C(6)H), 4.14 (s, 3H, OMe); ^{13}C NMR (176 MHz, DMSO-d_6) δ 163.95 (C(4)), 157.82 (C(7a)), 155.15 C(2), 132.83 C(6), 116.41 (C(4a)), 108.38 (C(7)), 54.59 (OMe); LCMS *m/z* (ESI$^+$) 244.9 ([M(^{79}Br)+H]$^+$, 100%), 247.0 ([M(^{81}Br)+H]$^+$, 100%) ; HRMS (ESI$^+$) $C_7H_5{}^{79}BrN_2OS^+$ ([M+H]$^+$) requires 244.9379; found 244.9378.

Alternative synthesis: 7-Bromo-4-chlorothieno[3,2-*d*]pyrimidine (**16**) was purchased from Fluorochem (076092). To a suspension of sodium methoxide (10.8 g, 200 mmol, 5.0 equiv) in 1,4-dioxane (80 mL) under nitrogen was added 7-bromo-4-chlorothieno[3,2-*d*]pyrimidine (**16**) (9.98 g, 40 mmol) in one portion. The resulting mixture was stirred at room temperature for 18 h. The solution was concentrated *in vacuo*

and the residue was diluted with water (400 mL) and extracted with ethyl acetate (3x200 mL). The combined organic phases were washed with brine, dried ($MgSO_4$) and concentrated *in vacuo* to give **5** (8.18 g, 84%) as pale crystals.

7-Cyclopropyl-4-methoxythieno[3,2-d]pyrimidine (**6**).

A 100 mL round-bottomed flask fitted with a condenser and magnetic stirrer was charged with 2-cyclopropyl-6-methyl-1,3,6,2-dioxazaborocane-4,8-dione (**11**) (1.93 g, 9.79 mmol, 1.5 equiv), 7-bromo-4-methoxy-thieno[3,2-*d*]pyrimidine (**5**) (1.6 g, 6.53 mmol), K_3PO_4 (8.3 g, 39.2 mmol, 6.0 equiv) and Pd[$(PPh_3)_2Cl_2$] (229 mg, 0.33 mmol, 5 mol%) and the flask purged with argon. Toluene (18 mL) and water (6 mL) were added, the reaction mixture was deoxygenated by sonicating under vacuum and back-filling with argon (repeat x3) and then heated at 100 °C with rapid stirring for 16 h. After such time, LCMS analysis showed complete conversion of bromide **5** to the cyclopropyl product **6**. Accordingly, the reaction mixture was cooled to room temperature, diluted with ethyl acetate (20 mL) and filtered through a pad of celite®, eluting with ethyl acetate (20 mL). The filtrate was washed with water (30 mL) and the aqueous phase back-extracted with ethyl acetate (2 × 30 mL). The organics were combined, dried (brine, Na_2SO_4), filtered and concentrated in vacuo to give a gum†. This was taken up in the minimum volume of toluene and purified by flash chromatography using a Biotage Isolera One 3.0 with a 50 g KP-Sil SNAP cartridge (linear gradient 0–20% ethyl acetate in cyclohexane) to give **6** (1.26 g, 6.13 mmol, 94%) as a white solid.

Mp 63-64 °C ; ν_{max} (neat) 3050 w, 3000 w, 2950 w, 1572 s, 1541 m, 1506 m, 1462 m; ^{1}H NMR (600 MHz, DMSO-*d*$_6$) δ 8.79 (s, 1H, C(2)H), 7.78 (s, 1H, C(6)H), 4.11 (s, 3H, OMe), 2.36 – 2.27 (m, 1H, CH), 1.03 – 0.90 (m, 4H, 2 x CH_2); ^{13}C NMR (151 MHz, DMSO-*d*$_6$) δ 163.98 (C(4)), 160.51 (C(7a)), 154.01 (C(2)), 139.51 (C(7)), 126.72 (C(6)), 117.21 (C(4a)), 54.17 (OMe), 9.15 (CH), 8.24 (2 x CH_2) LCMS *m/z* (ESI$^+$) 207.1 ([M+H]$^+$, 100%); HRMS (ESI$^+$) $C_{10}H_{10}N_2OS^+$ ([M+H]$^+$) requires 207.0587; found 207.0588.

When performed on larger scale (**5**: 9.3 g, 37.9 mmol), the reaction stalled after ca. 90% conversion and addition of extra catalyst Pd[$(PPh_3)_2Cl_2$] and/or **11** failed to drive the reaction to completion. We found the most efficient way to complete the reaction was to isolate the crude product (mostly **6**; designated by † above) and subject this material to a repeat reaction. This procedure gave **6** (7.4 g, 35.9 mmol, 95%) as an off-white solid.

6-Chloro-7-cyclopropyl-4-methoxythieno[3,2-d]pyrimidine (**7**).

Trial reactions (n = 2): 1-Chloro-1,2-benziodoxol-3-one (**12**) (514 mg, 1.82 mmol, 1.5 equiv) and 7-cyclopropyl-4-methoxy-thieno[3,2-*d*]pyrimidine **6** (250 mg, 1.21 mmol) were added to a 10 mL thick-walled reaction vial. The vessel was then sealed with a Teflon-lined crimp cap, *N,N*-dimethylformamide (2.5 mL) was added and the reaction mixture then heated to 50 °C for 16 h. The mixture was cooled to room temperature, diluted with dichloromethane (ca 5 mL) and then dry-loaded onto ISOLUTE® HM-N. The residue was then purified by column chromatography on silica gel (0-3% ethyl acetate in cyclohexane) to give 6-chloro-7-cyclopropyl-4-methoxythieno[3,2-*d*]pyrimidine **7** (260-280 mg, 1.02–1.14 mmol, 84–94%) as a white solid.

Mp 66-67 °C ; ν_{max} (neat) 3042 w, 3003 w , 1574 s, 1541 m, 1506 s, 1460 m, 1431 m; ^{1}H NMR (700 MHz, DMSO-d_6) δ 8.74 (s, 1H (C(2)H), 4.09 (s, 3H, OMe), 2.14 (tt, $^3J_{HH}$ = 8.7 Hz, $^3J_{HH}$ = 5.3 Hz, 1H, CH), 1.51 – 1.47 (m, 2H, 2 x CH_aH$_b$), 1.02 – 0.99 (m, 2H, 2 x CH$_a$$H_b$); ^{13}C NMR (176 MHz, DMSO-d_6) δ 162.79 (C(4)), 158.64 (C(7a)), 154.49 (C(2)), 133.89 (C(7)), 133.62 (C(6)), 114.64 (C(4a)), 54.35 (OMe), 9.82 (CH), 5.74 (2 x CH_2); LRMS *m/z* (ESI$^+$) 241.1 ([M(^{35}Cl)+H]$^+$, 100%), 243.1 ([M(^{37}Cl)+H]$^+$, 30%); HRMS (ESI$^+$) $C_{10}H_9{}^{35}ClN_2OS^+$ ([M+H]$^+$) requires 241.0202; found 241.0200.

Larger scale (5.0 g, 24.2 mmol) by parallel batch reactions: To each of five 20 mL thick-walled reaction vials was added 1-chloro-1,2-benziodoxol-3-one (**12**) (2.05 g, 7.27 mmol, 1.5 equiv) and 7-cyclopropyl-4-methoxy-thieno[3,2-*d*]pyrimidine **6** (1.0 g, 4.85 mmol). Each vessel was sealed with a Teflon-lined crimp cap, *N*,*N*-dimethylformamide (10 mL) was added and the mixture was heated at 50 °C for 24 h. The vessels were cooled to room temperature and the five reaction mixtures were then combined. This mixture was filtered through a plug of silica gel washing with ethyl acetate (ca 50 mL), the combined filtrates were concentrated under reduced pressure and the orange oil (ca. 11 g) was suspended in water (50 mL) and concentrated on a freeze-dryer overnight. The residue was purified by column chromatography on silica gel (1–2% ethyl actetate in cyclohexane) to give **7** (4.52 g, 18.8 mmol, 77%) as a white solid.

6-Chloro-7-cyclopropylthieno[3,2-d]pyrimidin-4(3H)-one (**8**).

Conc. hydrochloric acid (37%, 17 mL, 560 mmol) was added to 6-chloro-7-cyclopropyl-4-methoxy-thieno[3,2-*d*]pyrimidine (**7**) (3.4 g, 14.1 mmol) and the suspension warmed to 70 °C. The resultant pale yellow solution was heated at 70 °C for a further 16 h, the reaction was then cooled to room temperature, and concentrated under reduced pressure. The residue was dried by azeotroping with toluene (×3) and diethyl ether (×3) to afford 6-chloro-7-cyclopropylthieno[3,2-*d*]pyrimidin-4(3*H*)-one (**8**) (3.2 g, 14.1 mmol, quant.) as an off-white solid which was used without further purification.

Mp 231 °C (dec); ν_{max} (neat) 3007 br, 1645 s, 1597 s, 1531 m, 1447 m, 1414 m; ^{1}H NMR (700 MHz, DMSO-*d*$_6$) δ 12.68 (s, 1H, NH), 8.15 (d, $^3J_{HH}$ = 3.4 Hz, 1H, C(2)H), 2.04 (tt, $^3J_{HH}$ = 8.7 Hz, $^3J_{HH}$ = 5.3 Hz, 1H), 1.38 – 1.35 (m, 2H, 2 x C$\underline{H_a}$H$_b$).), 0.97 – 0.93 (m, 2H, 2 x CH$_a$$\underline{H_b}$); ^{13}C NMR (176 MHz, DMSO-*d*$_6$) δ 156.16 (C(4)), 154.94 (C(7a)), 146.96 (C(2)), 134.89 (C(6)), 132.45 (C(7)), 120.67 (C(4a), 9.73 (CH), 5.79 (2 x CH_2); LCMS *m/z* (ESI$^+$) 227.0 ([M(^{35}Cl)+H]$^+$, 100%), 229.0 ([M(^{37}Cl)+H]$^+$, 30%); HRMS (ESI$^+$) $C_9H_7ClN_2OS^+$ ([M+H]$^+$) requires 227.0040; found 227.0040.

7-Cyclopropyl-4,6-dichlorothieno[3,2-d]pyrimidine (**9**).

Phosphorus (V) oxychloride (26 mL, 279 mmol, 20 equiv) was added to 6-chloro-7-cyclopropylthieno[3,2-d]pyrimidin-4(3H)-one (**8**) (3.2 g, 14.1 mmol) and the mixture was heated to 90 °C for 16 h. The cooled mixture was then concentrated under reduced pressure and the residue azeotroped with dichloromethane (x 2). The flask was cooled (0 °C) and ice water (50 mL) was added. The resultant solid was collected *via* filtration, dissolved in dichloromethane (ca 50 mL), dried ($MgSO_4$) and then concentrated under reduced pressure to give 7-cyclopropyl-4,6-dichlorothieno[3,2-*d*]pyrimidine (**9**) (2.8 g, 11.4 mmol, 81%) as a light tan solid which was used without further purification.

LCMS *m/z* (ESI$^+$) 245.0 ([M(^{35}Cl)+H]$^+$, 100%), 247.0 ([M(^{37}Cl)+H]$^+$, 30%).

Methyl 2-((6-chloro-7-cyclopropylthieno[3,2-d]pyrimidin-4-yl)thio)acetate (**10**).

Methyl thioglycolate (**13**) (1.3 mL, 13.7 mmol, 1.2 equiv) was added cautiously to a solution of 4,6-dichloro-7-cyclopropylthieno[3,2-*d*]pyrimidine (**9**) (2.8 g, 11.4 mmol) and triethylamine (3.3 mL, 24.0 mmol, 2.1 equiv) in methanol (100 mL) at 0 °C. The ice bath was removed and the mixture was warmed to room temperature and then stirred for 16 h. The reaction mixture was concentrated under reduced pressure and the residue purified by column chromatography on silica gel (0–10% ethyl acetate in cyclohexane) to give methyl 2-((6-chloro-7-cyclopropylthieno[3,2-*d*]pyrimidin-4-yl)thio)acetate (**10**) (3.0 g, 9.6 mmol, 84%) as a white solid.

Mp 79-80 °C; ν_{max} (neat) 3007 w, 2990 w, 2947 w, 1740 m, 1647 m), 1597 m, 1551 m, 1514 m, 1489 m, 1447 m; ^{1}H NMR (700 MHz, DMSO-*d*$_6$) δ 8.91 (s, 1H, C(2)H), 4.31 (s, 2H, SCH_2), 3.66 (s, 3H, OMe), 2.15 (tt, $^3J_{HH}$ = 8.7 Hz, $^3J_{HH}$ = 5.3 Hz, 1H, CH), 1.50 – 1.45 (m, 2H, 2 x CH$_a$H$_b$), 1.03 – 0.99 (m, 2H, 2 x CH$_a$H$_b$); ^{13}C NMR (176 MHz, DMSO-*d*$_6$) δ 168.54 (C(9)), 160.60 (C(4)), 155.89 (C(7a)), 153.71 (C(2)H), 134.15 (C(6) or (C(7)), 134.00 (C(6) or (C(7)), 125.49 (C(4a)), 52.58 (OMe), 31.01 (SCH_2), 9.77 (CH), 5.80 (2 x CH_2); LCMS *m/z* (ESI$^+$) 315.0 ([M(^{35}Cl)+H]$^+$, 100%), 316.9 ([M(^{37}Cl)+H]$^+$, 30%); HRMS (ESI$^+$) $C_{12}H_{11}ClN_2O_2S_2^+$ ([M+H]$^+$) requires 315.0023; found 315.0024.

2-((6-Chloro-7-cyclopropylthieno[3,2-d]pyrimidin-4-yl)thio)acetic acid, **LP-922056** (**1**).

[CAS Registry Number: 1365060-22-5; SMILES: ClC1=C(C2CC2)C3=C(S1)C(SCC(O)=O)=NC=N3]

A solution of NaOH (770 mg, 19.3 mmol, 2.0 equiv) in water (18 mL) was added to a stirred solution of methyl 2-((6-chloro-7-cyclopropylthieno[3,2-*d*]pyrimidin-4-yl)thio)acetate (**10**) (3.0 g, 9.6 mmol) in THF (20 mL) at 0 °C and the mixture was stirred for 1 h. Solvents were evaporated under reduced pressure, the residue suspended in water (1 mL), cooled to 0 °C and then acidified with aq. HCl (1 M) to pH 2–3. The resultant slurry was stirred for 30 minutes and the precipitate collected by filtration, washed with water (ca 20 mL), and then dried in vacuo to give 2-((6-chloro-7-cyclopropylthieno[3,2-*d*]pyrimidin-4-yl)thio)acetic acid (**1**) (2.9 g, 98%) as a white solid (purity > 99%).

Mp 207 °C (dec); ν_{max} (neat) 2800 br, 1717 m, 1549 m, 1501 m, 1419 m; ^{1}H NMR (700 MHz, DMSO-d_6) δ 12.97 (s, 1H, OH), 8.92 (s, 1H, C(2)H), 4.23 (s, 2H, SCH_2), 2.17 – 2.13 (m, 1H, CH), 1.50 – 1.48 (m, 2H, 2 x $C\underline{H_a}H_b$), 1.04 – 1.00 (m, 2H, 2 x $CH_a\underline{H_b}$); ^{13}C NMR (176 MHz, DMSO-d_6) δ 169.2 (CO_2H), 161.0 (C(4)), 155.8 (C(7a)), 153.7 (C(2)), 134.0 (s, C(6) or C(7)), 134.0 (s, C(6) or C(7)), 125.5 (C(4a)), 31.5 (SCH_2), 9.8 (CH), 5.8 (2 x CH_2); LRMS *m/z* (ESI$^+$) 301.0 ([M(^{35}Cl)+H]$^+$, 100%), 302.9 ([M(^{37}Cl)+H]$^+$, 30%); HRMS (ESI$^+$) $C_{11}H_9ClN_2O_2S_2^+$ ([M+H]$^+$) requires 300.9867; found 300.9866.

LP-922056 (**1**) will be available from Tocris Bioscience (6691).

Animal testing declaration:

In vivo mouse pharmacokinetic data was generated at Pharmidex Pharmaceutical Services Ltd, UK and all animal experimental protocols have been approved by the UK Government Home Office and carried out in accordance with the guidelines of the Animals (Scientific Procedures) Act (1986).

Table S1: Study design summary

Compound:

Compound ID	MW (salt)	MW (free base)
LP-922056	-	300.8

Species (strain, sex): Mouse (CD1, ♂) **n =** 3 /timepoint (Total = 21)

Animal group numbers:

PO 1	PO 2	PO 3

Fed/Fasted: Fed

Dose Route: Oral

	LP-922056
**Dose Level:**	10 mg/kg
**Dose Concentration:**	1.0 mg/mL
**Dose Volume:**	10.0 mL/kg

Formulation: Suspension in 0.1% Tween 80 in water

Sampling: Terminal

Animal Husbandry: Animals housed in pre-assigned housing cages until sampling. Appropriate samples were taken at the defined time points and stored immediately at -20°C.

Matrices Collected: Plasma Brain

Matrix: Plasma

**Timepoints (hr):**	0.17	0.50	1.0	2.0	5.0	7.5	24

Sample Preparation: Protein precipitation with acetonitrile.

Matrix: Brain

**Timepoints (hr):**	0.17	0.50	1.0	2.0	5.0	7.5	24

Sample Preparation: Protein precipitation with acetonitrile.

Note: Sampling time points adjusted from nominal.

Sample Analysis: UHPLC - TOF mass spectrometry using electrospray ionisation.

Table S2: Plasma concentrations

In Vivo Pharmacokinetic Assessment of LP-922056 Dosed Orally at 10 mg/kg

[LP-922056] in Plasma					
Time (hr)	[LP-922056] ng/mL				
	PO-1	PO-2	PO-3	mean	SD
0.17	21449	19884	15987	19107	2813
0.50	31844	27416	20409	26557	5766
1.0	38201	32492	33987	34893	2960
2.0	36456	37011	32664	35377	2366
5.0	25283	21622	23771	23559	1839
7.5	16644	7052	5346	9681	6090
24	3065	4139	4898	4034	921

<LLOQ = Below Limit of Quantification, 100 ng/mL
NR = No Result

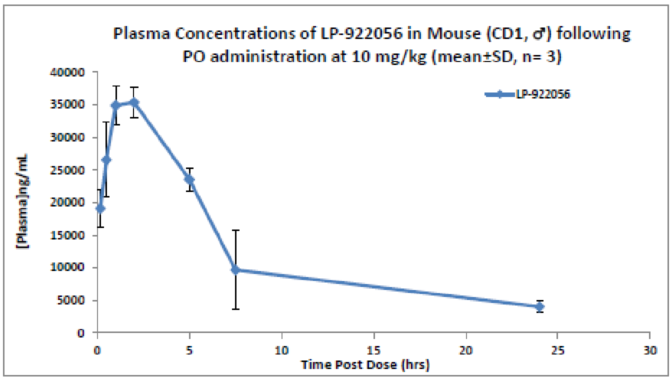

PK Parameter	Mean Value
$t_{1/2}$	8.8 hr
T_{max}	2.0 hr
C_{max}	35377 ng/mL
AUC_{last}	302754 hr*ng/mL
AUC_{all}	302754 hr*ng/mL
AUC_{inf}	354144 hr*ng/mL

Parameters determined using Winnonlin Version 7.0

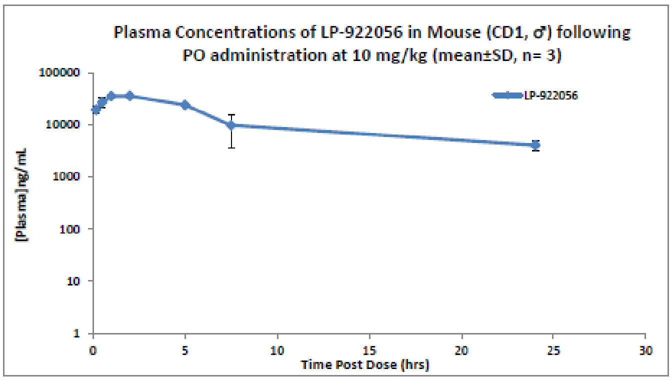

Table S3: Brain concentrations

In Vivo Pharmacokinetic Assessment of LP-922056 Dosed Orally at 10 mg/kg

[LP-922056] in Brain					
Time (hr)	[LP-922056] ng/g				
	PO-1	PO-2	PO-3	mean	SD
0.17	415	297	188	300	114
0.50	334	269	221	274	57.1
1.0	499	449	555	501	53.0
2.0	445	512	551	503	53.5
5.0	454	283	284	340	98.5
7.5	165	76.7	47.0	96.2	61.3
24	<LLOQ	41.9	44.9	35.6	13.6

<LLOQ = Below Limit of Quantification, 40 ng/g
NR = No Result

Mean and STDEV calculated using 0.5 x LLOQ where individual concentration <LLOQ

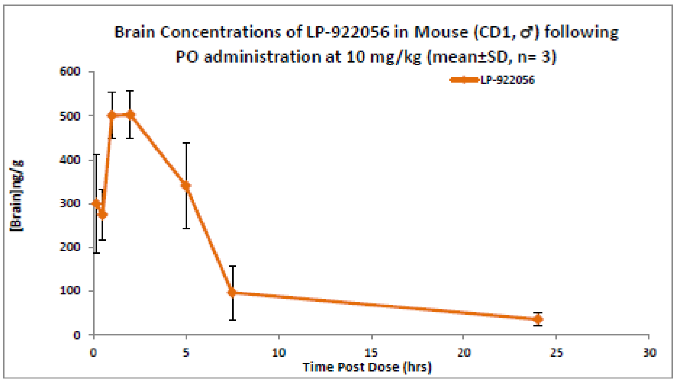

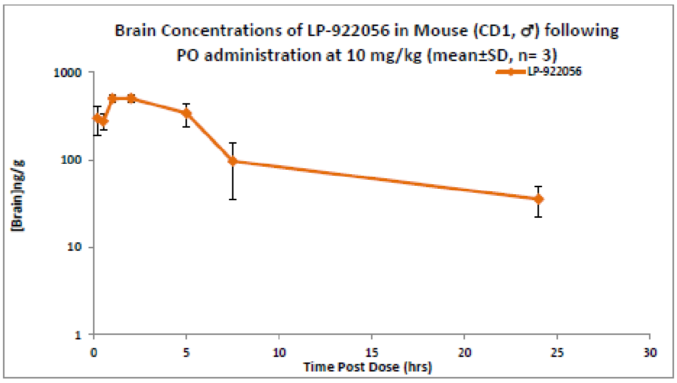

PK Parameter	Mean Value
$t_{1/2}$	7.1 hr
T_{max}	2.0 hr
C_{max}	503 ng/g
AUC_{last}	3714 hr*ng/g
AUC_{all}	3714 hr*ng/g
AUC_{Inf}	4078 hr*ng/g

Parameters determined using Winnonlin Version 7.0

LP-922056 Brain:Plasma Ratio					
Time (hr)	brain:plasma ratio				
	PO-1	PO-2	PO-3	mean	SD
0.17	0.02	0.01	0.01	0.02	0.00
0.50	0.01	0.01	0.01	0.01	0.00
1.0	0.01	0.01	0.02	0.01	0.00
2.0	0.01	0.01	0.02	0.01	0.00
5.0	0.02	0.01	0.01	0.01	0.00
7.5	0.01	0.01	0.01	0.01	0.00
24	-	0.01	0.01	0.01	0.00

Table S4: Profiles of amides 17a–r

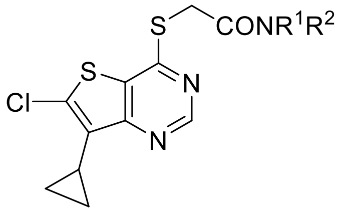

17a-r

Compound	NR^1R^2	**Notum** IC_{50} (nM)[a]	**MLM** Cl_i (µL/min/mg)[b]	**MDCK-MDR1** AB/BA P_{app} (x10^{-6} cm/s)[b] Extraction Ratio (ER)
Our project criteria	-	*< 10*	*< 10*	*> 10 / -*<br>*< 3*[c]
cf. **1**	-	1.1 ± 0.4	1	
**17a**	$-NMe_2$	7.5 ± 12.4		
**17b**	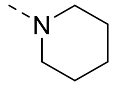	91 ± 67		
**17c**	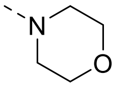	18 ± 8.7	>500	40/38<br>0.95
**17d**	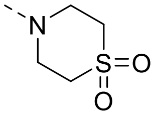	48		
**17e**	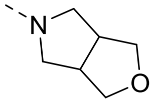	10 ± 2.7	>500	
**17f**	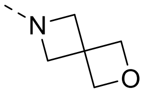	10 ± 3.8	100	
**17g**	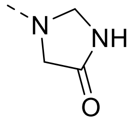	1.5 ± 0.1	19	3.8/14<br>3.7
**17h**	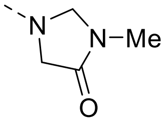	1.5 ± 0.2	45	6.0/13<br>2.2
**17i**	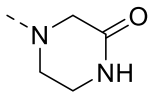	7.1 ± 4.1	24	7.9/65<br>8.2

**17j**	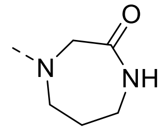	36 ± 9.4		
**17k**	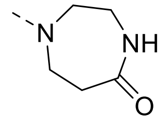	49 ± 23		
**17l**	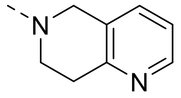	4.6 ± 1.8		
**17m**	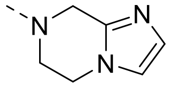	2.7 ± 0.4	290	21/25<br>1.2
**17n**	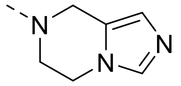	2.5 ± 0.1	49	9.0/31<br>3.4
**17o**	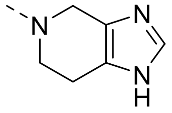	2.3 ± 0.6		
**17p**	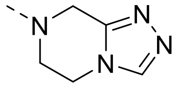	1.2 ± 0.3	29	0.95/93<br>98
**17q**	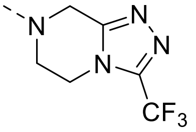	8.0 ± 0.7	190	12/34<br>2.8
**17r**	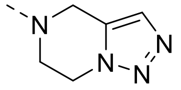	2.4 ± 0.1	110	26/28<br>1.1

a Test compounds (dispensed to give 10 point concentration-response-curves) were incubated with Notum(81-451 Cys330Ser) and trisodium 8-octanoyloxypyrene-1,3,6-trisulfonate (OPTS) as the substrate for 1 h, and fluorescence recorded; an inhibitor of Notum would suppress fluorescence by binding to Notum and preventing hydrolysis of OPTS. All values are geometric mean ± s.d. of n = 2-8 experiments quoted to 2 s.f., except **17d** (n = 1). Screening data only passed the quality control criteria if the screening plates demonstrated a Z' > 0.5 and the Notum inhibition activity of the positive control was within acceptable limits (IC_{50} 0.6 – 1.1 nM). Differences of <2-fold should not be considered significant [3].

b MLM and MDCK-MDR1 studies reported in this work were independently performed by GVK Biosciences (Hyderabad, India. https://www.gvkbio.com/discovery-services/biology-services/dmpk-services/) or Cyprotex (Macclesfield, UK. https://www.cyprotex.com/admepk).

c See Reference 4.

SI References

1. Mitchell, I. S.; Spencer, K. L.; Stengel, P.; Han, Y.; Kallan, N. C.; Munson, M.; Vigers, G. P. A.; Blake, J.; Piscopio, A.; Josey, J.; Miller, S.; Xiao, D.; Xu, R.; Rao, C.; Wang, B.; Bernacki, A. L. Akt protein kinase inhibitors. WO Patent 2005/051304, June 9, 2005.

2. Atheral, J. F.; Hough, T. L.; Lindell, S. D.; O'Mahony, M. J.; Parsons, J. H.; Saville-Stones, E. A. Fungicides. US Patent 6,432,964, August 13, 2002.

3. Atkinson, B. N.; Steadman, D.; Zhao, Y.; Sipthorp, J.; Vecchia, L.; Ruza, R. R.; Jeganathan, F.; Lines, G.; Frew, S.; Monaghan, A.; Kjaer, S.; Bictash, M.; Jones, E. Y.; Fish, P. V. *Med. Chem. Comm.* **2019**, *10*, 1361-1369.

4. Hitchcock, S. A. *J. Med. Chem.* **2012**, *55*, 4877-4895.
