# Supplementary material for: An improved, scalable synthesis of Notum inhibitor LP-922056 using 1-chloro-1,2-benziodoxol-3-one as a superior electrophilic chlorinating agent
Source: Beilstein J Org Chem. 2019 Nov 19;15:2790–7. doi: 10.3762/bjoc.15.271 (PMC6880826; doi:10.3762/bjoc.15.271)
Supplement: File 3 — Raw NMR data files for compound LP-922056. [file Beilstein_J_Org_Chem-15-2790-s003.zip › NW-0016-031-FD/10/pdata/1/email_NW-0016-031-FD_10_1.pdf]

PROTON.ucl DMSO {C:\700} alz 1

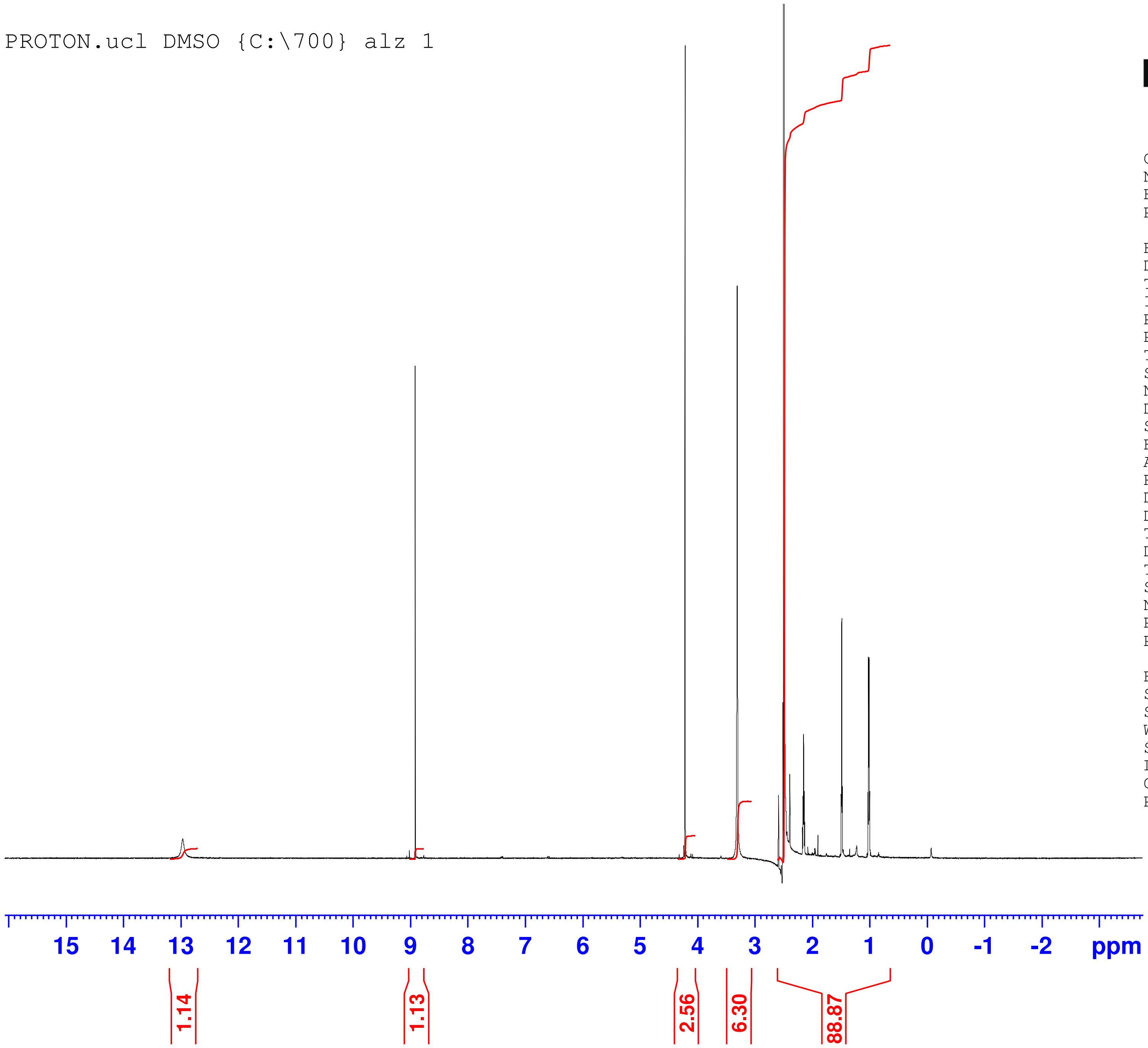

Current Data Parameters

NAME	NW-0016-031-FD
EXPNO	10
PROCNO	1

F2 - Acquisition Parameters

Date_	20180110
Time	13.32 h
INSTRUM	spect
PROBHD	Z123726_0032 (
PULPROG	zg30
TD	111110
SOLVENT	DMSO
NS	32
DS	2
SWH	13888.889 Hz
FIDRES	0.250003 Hz
AQ	3.9999599 sec
RG	101
DW	36.000 usec
DE	6.64 usec
TE	298.2 K
D1	1.00000000 sec
TD0	1
SFO1	700.3543247 MHz
NUC1	1H
P1	16.00 usec
PLW1	12.20199966 W

F2 - Processing parameters

SI	131072
SF	700.3500126 MHz
WDW	EM
SSB	0
LB	0.30 Hz
GB	0
PC	1.00
