# Supplementary material for: An improved, scalable synthesis of Notum inhibitor LP-922056 using 1-chloro-1,2-benziodoxol-3-one as a superior electrophilic chlorinating agent
Source: Beilstein J Org Chem. 2019 Nov 19;15:2790–7. doi: 10.3762/bjoc.15.271 (PMC6880826; doi:10.3762/bjoc.15.271)
Supplement: File 3 — Raw NMR data files for compound LP-922056. [file Beilstein_J_Org_Chem-15-2790-s003.zip › NW-0016-031-FD/11/pdata/1/email_NW-0016-031-FD_11_1.pdf]

C13CPD.ucl DMSO {C:\700} alz 1

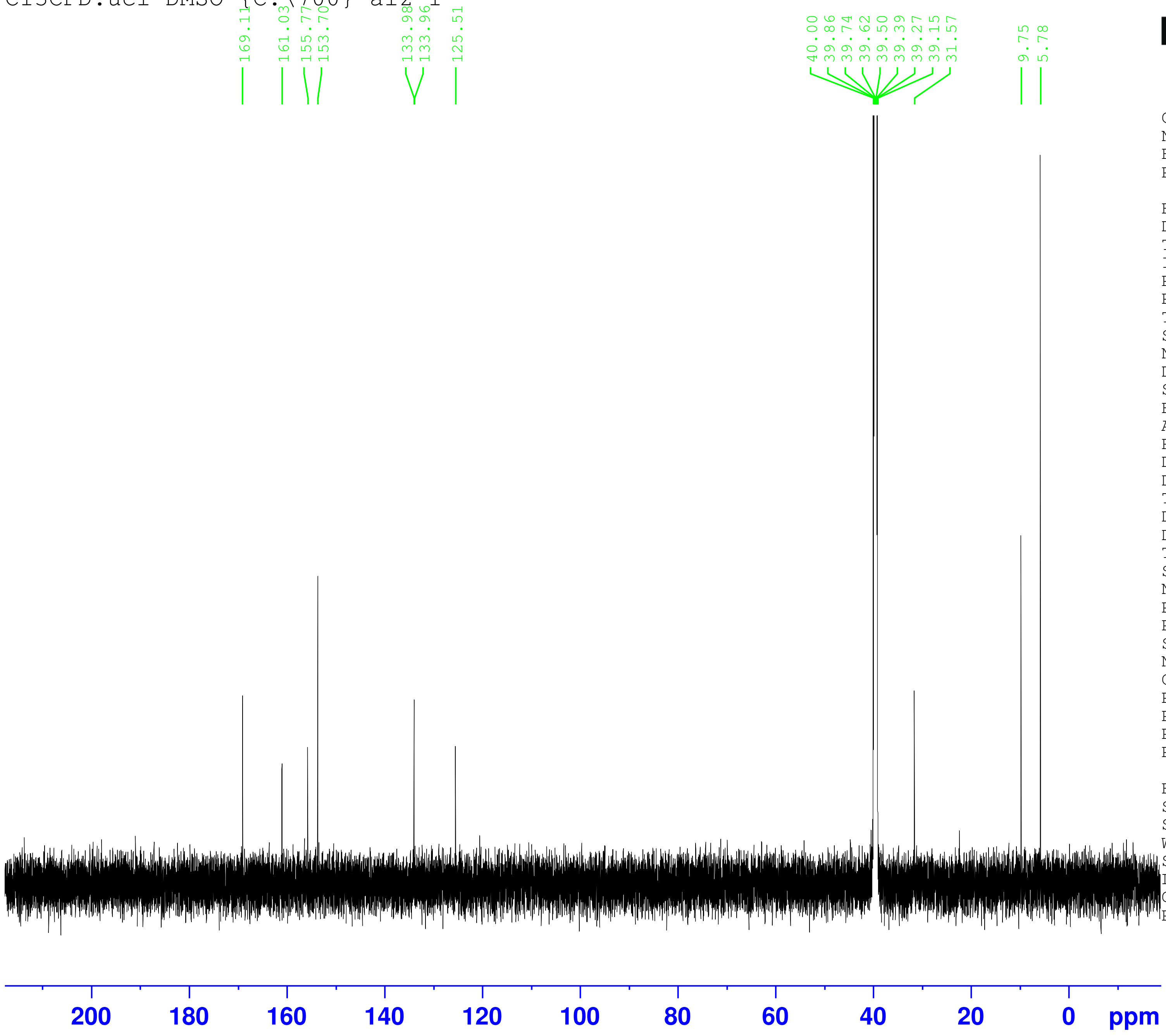

BRUKER

Current Data Parameters
NAME NW-0016-031-FD
EXPNO 11
PROCNO 1

F2 - Acquisition Parameters
Date_ 20180111
Time 5.35 h
INSTRUM spect
PROBHD Z123726_0032 (
PULPROG zgpg30
TD 65536
SOLVENT DMSO
NS 2048
DS 4
SWH 41666.668 Hz
FIDRES 1.271566 Hz
AQ 0.7864320 sec
RG 101
DW 12.000 usec
DE 7.48 usec
TE 298.2 K
D1 2.00000000 sec
D11 0.03000000 sec
TD0 1
SFO1 176.1207579 MHz
NUC1 13C
P1 12.00 usec
PLW1 64.79000092 W
SFO2 700.3528014 MHz
NUC2 1H
CPDPRG[2 waltz16
PCPD2 65.00 usec
PLW2 12.20199966 W
PLW12 0.73931998 W
PLW13 0.37228000 W

F2 - Processing parameters
SI 131072
SF 176.1032362 MHz
WDW EM
SSB 0
LB 1.00 Hz
GB 0
PC 1.40
